# Evaluation of Infection Control Practices and Washing Hand Behaviours in Dental Hygiene Students

**DOI:** 10.3290/j.ohpd.c_1809

**Published:** 2025-02-18

**Authors:** Sinan Yasin Ertem

**Affiliations:** a Sinan Yasin Ertem Associate Professor, Department of Oral and Maxillofacial Surgery, School of Dentistry, Ankara Yildirim Beyazit University, Ankara, Turkey. Researched and wrote the paper.

**Keywords:** dental hygiene, hand wash, infection

## Abstract

**Purpose:**

The aim of this study was to evaluate the behavioural habits, basic knowledge and handwashing habits of dental hygiene students regarding infection control procedures.

**Materials and Methods:**

60 dental hygiene students answered 26 questions and one open-ended question on infection control procedures between May and June 2023. The questions were designed to assess dental hygiene students’ attitudes about infection control protocols and handwashing practices.

**Results:**

According to the results of the questionnaire, it was observed that students had a high awareness of the necessity of handwashing but a low awareness of other infection control methods. The main reasons for not washing hands were found to be lack of time and forgetfulness due to workload and lack of adequate handwashing areas.

Conclusions: Providing adequate protective equipment, reducing the high workload that may cause forgetfulness and reducing physical deficiencies such as increasing the number of handwashing units are measures that can increase washes. It is important to increase the level of education and knowledge of healthcare professionals in line with current information on handwashing and infection prevention procedures.

Infections can be transmitted in a variety of ways, including direct contact with blood, oral fluids, or other secretions during dental treatment procedures. Transmission can occur through indirect contact with contaminated instruments, surgical equipment, and peripheral surfaces; with droplet splashes, or with coming in contact with airborne microparticles in the aerosols of oral and respiratory fluids.^5,13^ Studies have reported that respiratory viruses are transmitted by direct or indirect contact between people or macro-micro saliva droplets.^2^

Some of the infection control procedures were limited to general rules such as completely replacing contaminated clothing used in clinical conditions, not using jewellery, preventing saliva contamination in the maxillofacial area during treatment, and washing hands. It is very important that practitioners and educators in the dentistry community pioneer new graduates in changing their attitude toward infection control practices. For the dental treatment of patients with infectious diseases, it is vital to scientifically define the transmission paths and educate dental professionals via diagrams. In addition, certain standards must be established for the necessary protective equipment. Hand hygiene is the most critical measure to prevent infection for both patients and healthcare professionals.^8^ Failure to comply with hand hygiene practices can lead to the spread of health-related infections, resistant organisms, or infectious viruses.^3^ Training can be achieved at all levels and can be the first step in changing the attitudes and practice habits of dental professionals. Dental workers in high-risk groups state that they have a reluctance to treat patients, especially during epidemic periods.^7^ Defining the standards on infection control and providing training on this subject is an indispensable element of changing the attitudes and practices of dental professionals. It is important to update these infection control standards rapidly, as they may have different infectious properties in the mutation of viruses.

The aim of this study was to evaluate the behavioural habits of dental hygiene students regarding infection control procedures and to suggest an update in the infection prevention protocols of dental professionals based on these evaluations.

## MATERIALS AND METHODS

The study was conducted with the participation of students enrolled in the Dental Hygiene Programme at Ankara Yildirim Beyazit University in Ankara, Turkey. The students included in the study were eligible to enrol in this department without any geographical or ethnic restrictions, based on their scores and preferences in a nationally administered central examination following high school graduation. Our current cohort comprises students who entered the programme after completing the same standard basic education. None of the students had received any vocational training prior to entering the programme. Students coming from all regions of Turkey were included in the study without any restrictions. The students complete their education through a two-year programme after high school and graduate as dental hygienists. The research was carried out from May to June 2023 among dental hygiene students to evaluate their approach to infection control practices. The approach of the researchers was ensured to be non-coercive, and written informed consent was obtained from all participants before the questionnaire was completed. Ethical approval for this study was obtained from Ankara Yildirim Beyazit University Ethics Committee (2019-227/16). Before the questionnaire was administered, students were informed about the nature and content of the study.

The study group consisted of a total of 60 students, of which 38 were first-year students and 22 second-year Oral and Dental Health programme students. There was a favourable gender balance among the groups included in the study. 55% (33 females) of the students identified as female, while 45% (27 males) identified as male. This gender diversity facilitates an inclusive assessment of the knowledge and behaviour levels of all students concerning infection control procedures. The average age of the participants was 19.5 ± 1.3 years. In order to evaluate hand hygiene habits, a questionnaire consisting of 27 questions was applied to the students. Questionnaire questions were written in a clear and intelligible language. In the survey, the questions were addressed under the following main headings:

Questions about the frequency and duration of handwashing application. Answer choices included never, occasionally, and always.
Questions about measures to be taken in terms of infection control. Answer choices included Yes and No.
Questions regarding the self-protection and control of the dental assistant during treatment procedures. Answer choices included Yes and No.
A question to identify the reasons preventing handwashing was asked, with five possible reasons.
An open-ended question regarding infection control procedures has been posed.

## RESULTS

The numbers and rates of the correct answers to the questionnaire questions directed to oral and dental assistant students are shown in Table 1 and Table 2. In questions 1, 2, 3, 4, which are related to handwashing behaviour before and after a dental treatment procedure, the majority of those who answered questions stated that it is necessary to wash hands sometimes or always. In questions 5 and 6, nearly all of the participants stated that they always wash their hands in case of contamination. All but one of those who answered questions 9, 10, 11, and 12 related to the basic theoretical knowledge about the effects of handwashing on the spread of infection answered all four questions correctly. Regarding the necessity of wearing a mask for question 13, all but one of the participants said that it was necessary. The lowest correct answer rate was for the questions 14, 15, 16, and 19. In these questions, the number of correct answers was 16 students (26.67%) for the question related to wearing medical protective glasses during dental treatment procedures, 14 students (23.33%) for the question related to eating and drinking in clinical settings, 17 (28.33%) for the question related to wearing jewellery while working close to the patient, and was 18 (30.00%) for the question related to wearing a medical protective visor in clinical settings. Regarding preventive basic measures, the questions numbered 17, 20, and 22 were answered with an accurate answer rate of over 90%. More than half of the participants answered questions 21 and 23 correctly, and only half of the participants answered questions 24, 25, and 26 correctly.

**Table 1:** Survey questions asked to dental hygiene students to evaluate general behaviour on infection control

1. I wash my hands before wearing medical gloves	3 (5.00%)	35 (58.33%)	22 (36.67%)
2. I wash my hands after removing medical gloves	13 (21.67%)	23 (38.33)	24 (40.00%)
3. I wash my hands if the medical gloves tear	4 (6.67%)	22 (38.67%)	34 (56.67%)
4. I wash my hands after leaving a dental treatment procedure	5 (8.33%)	16 (26.67%)	39 (65.00%)
5. I wash my hands when I consider them contaminated	None	1 (1.67%)	59 (98.33%)
6. I wash my hands when they are visibly contaminated	None	3 (5.00%)	57 (95.00%)
7. I wash my hands before having lunch	None	18 (30.00%)	42 (70.00%)
8. I wash my hands after using the bathroom	None	None	60 (100%)

**Table 2:** Survey questions asked dental hygiene students about the protective measures which are included in infection control protocols

9. Hand hygiene prevents infection from spreading to patients	59 (98.33%)	1 (1.67%)
10. Hand hygiene prevents infection from spreading to the family members of medical staff	59 (98.33%)	1 (1.67%)
11. Hand hygiene prevents infection from spreading to medical staff	59 (98.33%)	1 (1.67%)
12. I change my medical gloves between patients	59 (98.33%)	1 (1.67%)
13. I wear protective medical masks during dental treatment procedures	59 (98.33%)	1 (1.67%)
14. I wear my glasses during dental treatment procedures	44 (73.33%)	16 (26.67%)
15. I eat and drink in the clinic	14 (23.33%)	46 (76.67%)
16. I wear various jewellery during dental treatment procedures	17 (28.33%)	43 (71.67%)
17. I wear medical gloves before touching patients’ face, skin, and intraoral area	58 (96.67%)	2 (3.33%)
18. I wash my hands before and after dental treatment procedures	49 (81.67%)	11 (18.33%)
19. I wear protective visor during dental treatment procedures	18 (30.00%)	42 (70.00%)
20. I wash my hands after touching patients’ bodily fluids	55 (91.67%)	5 (8.33%)
21. I wear medical protective clothing during dental treatment procedures to protect myself	38 (63.33%)	22 (36.67%)
22. I think wearing N95-FFP2 surgical masks during dental procedures is necessary	55 (91.67%)	5 (8.33%)
23. After injection processes, I bend the needles and dispose throw them into the medical waste bin	42 (70.00%)	18 (30.00%)
24. I check patients’ blood for infectious diseases before dental treatment procedures	26 (43.33%)	34 (56.67%)
25. I check any medical equipment for sterilisation before using them for dental treatment operations using the sterilisation indicator strips	31 (51.67%)	29 (48.33%)
26. I inform my patients about viruses and recommend them to get vaccinated	25 (41.67%)	35 (58.33%)

In the study, the questions in which we evaluate the reasons for washing hands less are shown in Table 3. The most common reasons for not washing hands were found to be forgetfulness due to workload with 34 students (56.67%), lack of adequate washing areas with 17 students (28.33%), and lack of time with 14 students (23.33%). Among the least stated reasons were considering washing hands as a waste of time with 6 students (10%), and skin irritation and allergies with 2 students (3.3%).

**Table 3:** Survey questions asked to dental hygiene students on the reasons of not washing hands (multiple answers are allowed.)

I consider washing hands as a waste of time.	6 (10.00%)
There is a lack of adequate handwashing areas.	17 (28.33%)
I forget washing my hands due to being busy.	34 (56.67%)
I do not have time for washing my hands due to being busy.	14 (23.33%)
I get skin irritations and allergies.	2 (3.33%)

When the students were asked about where they learned their knowledge about the infection control protocols in an open-ended question, 6 students did not answer and 54 students (%90) stated the training from their faculty as their source of knowledge.

## DISCUSSION

Handwashing is one of the most effective ways to reduce the spread of many infections in dentistry.^4^ The chain of infection requires a host, a pathogen with sufficient infectivity and number to cause an infection, and a path through which the pathogen can enter the host. Effective infection control strategies are meant to break one or more of these links in the chain, thereby preventing infection. It is known that handwashing plays an important role in infection control. When the results are examined, it is seen that the questions about handwashing, protective clothing, and basic infection control procedures are largely answered correctly. The survey shows that most of the participants have a high knowledge of the benefits of hand hygiene. Omogbai et al^11^ and Naik et al^10^ reported similarly highly accurate behaviours in accordance with infection control procedures as in this study. The high percentage of correct answers in the survey is encouraging and indicates that most hygiene professionals are willing to perform hand hygiene in their practice.

Handwashing is a very important indicator of the safety and quality of healthcare, as there is substantial evidence that demonstrates the correlation between good hand hygiene practices and low healthcare-related infection rates, and its ability to significantly reduce the number of microorganisms that can cause various infective diseases between patients and healthcare providers.^14^ Glove-wearing by dental personnel is a key element of cross-infection control in oral surgery. Hands are considered to be the primary source of infection and potentially infected blood may be localised under the finger and fingernails. Unless meticulous mechanical cleaning is performed, it can be difficult to remove the contaminated material, especially from the subungual and nail fold areas. In their study, El-Adawi et al^4^ reported that participants washed their hands before using gloves (27%) after gloves (73.9%), after torn gloves (69.6%), when they thought that hands were contaminated (83.5%), and before lunch (89.6%).

When the questionnaire questions in the study were analysed, the rates of handwashing habits before and after surgery were lower than the rates when the hands were considered dirty. Here, it is seen that awareness about the importance of washing hands before and after a dental treatment procedure should be raised during training. The questions (14, 15, 16, 19) related to conditions that we think will indirectly affect the spread of the infection such as eating and drinking in the clinic, using various jewellery while in the treatment procedure and glasses and visors that do not have protective features in daily life have considerably low correct answer rate. About half of the participants answered questions 24, 25 and 26 correctly, which included examining the laboratory test results before the treatment, checking the indicators of the materials to be sterilised, informing patients about the virus and making vaccination recommendations. In these questions, a lower rate of correct response was obtained since the precautions asked in these questions would have secondary effects on infection control.

There are several known factors affecting compliance with hand hygiene rules, such as lack of time, high patient workload, priority needs of patients, forgetfulness, lack of knowledge about the importance of hand hygiene in preventing cross-infection, and poor access to handwashing equipment. Factors such as lacking the habit of using hand hygiene products or having skin irritation as a result of handwashing have also been reported.^9^ In the study of Adawi et al,^4^ it was stated that lack of time and facilities are the most common obstacles for hand hygiene. In addition, Omogbai et al^11^ showed that the main obstacles to ensuring regular hand hygiene are the lack of adequate washing areas, forgetfulness, and lack of time. Lack of time was stated by Barrett et al^1^ as an obstacle to hand hygiene among dentists. Naik et al^10^ reported that skin irritation and dryness after handwashing, lack of sinks, and lack of time are the leading causes behind the lack of handwashing procedures. In this study, forgetfulness (56.67%) caused by excessive workload, insufficient handwashing areas (28.33%), and lack of time (23.33%) were found as the primary reasons, which complies with the literature. Students who stated skin irritation and allergies as a cause (3.3%) are also reported. However, the number of students complaining about this issue was lower compared to other studies. The low number of students (10%) who consider handwashing as unnecessary, shows that the level of awareness on the importance of washing hands is sufficient. Considering the primary reasons given by the students, we think that students’ workload should be lowered, and the number of handwashing areas should be increased in order to promote handwashing behaviours.

Strict adherence to standard precautions by all dental professionals is of paramount importance in the prevention of infectious diseases. Despite the great importance attached to standard infection control procedures, it has been reported that very few dental professionals adhere to these procedures in their clinical practice.^12^ Even in dental schools, it has been reported that future dentists do not always adhere to these procedures as required.6 The fact that all of the students participating in our study mentioned faculty education as a source of information about infection control emphasises the importance of education in this regard. Therefore, we think that appropriate education and training will also be effective in increasing the level of awareness of infection prevention procedures. Education in the field of dentistry can play an essential role in helping employees adopt adequate knowledge and attitudes about infection control measures.

## CONCLUSION

Assessing dental professionals’ adherence to infection control procedures based on their handwashing habits in the clinic is essential for taking effective preventive measures against possible infection. The most critical components that can affect infection control in dental practice are the adequacy of protective equipment, physical facilities, and the level of knowledge of dental professionals on the latest infection control procedures. Eliminating the lack of training and physical inadequacies in the clinic that may prevent complete washing procedures in the clinic are among the measures to reduce possible contamination. Although students’ awareness of handwashing is high, the same cannot be said for their awareness of other infection prevention methods. Therefore, there is a need to increase training in handwashing and general infection prevention procedures in line with clearly defined standards. In addition, necessary personal protective equipment should be provided, and physical barriers to infection control activities should be removed, such as increasing the number of handwashing units.
